# Self-aligned heterogeneous quantum photonic integration

**DOI:** 10.1038/s41377-026-02339-w

**Published:** 2026-07-15

**Authors:** Kinfung Ngan, Yeeun Choi, Chun-Chieh Chang, Dongyeon Daniel Kang, Shuo Sun

**Affiliations:** 1 JILA and Department of Physics, University of Colorado Boulder, Colorado, USA; 2 Center for Quantum Technology, Korea Institute of Science and Technology (KIST), Seoul, Republic of Korea; 3 KU-KIST Graduate School of Converging Science and Technology, Korea University, Seoul, Republic of Korea; 4 Center for Integrated Nanotechnologies, Los Alamos National Laboratory, Los Alamos, NM USA; 5 Division of Quantum Information, KIST School, Korea University of Science and Technology (UST), Seoul, Republic of Korea

**Keywords:** Quantum optics, Single photons and quantum effects, Nanophotonics and plasmonics

## Abstract

Integrated quantum photonics holds significant promise for scalable photonic quantum information processing, quantum repeaters, and quantum networks, but its development is hindered by the mismatch between materials hosting high-quality quantum emitters and those compatible with mature photonic technologies. Heterogeneous integration offers a potential solution to this challenge, yet practical implementations have been limited by inevitable insertion losses at material interfaces. Here, we present a self-aligned heterogeneous quantum photonic integration approach that enables near-unity coupling efficiency at the interface. To showcase our approach, we demonstrate Purcell enhancement of a silicon vacancy (SiV) center in diamond induced by a heterogeneous photonic crystal cavity defined by titanium dioxide (TiO_2_), as well as optical spin control and readout via a TiO_2_ photonic circuit. We further show that, when combined with inverse photonic design, our approach enables efficient and broadband collection of single photons from a color center into a heterogeneous waveguide. Our approach is not restricted to SiV centers or TiO_2_; it has the potential to be broadly applied to integrate diverse solid-state quantum emitters with thin-film photonic devices where conformal deposition is possible. Together, these results establish a practical route to scalable quantum photonic integrated circuits that combine high-quality quantum emitters with technologically mature photonic platforms.

## Abstract (web summary)

A self-aligned heterogeneous photonic integration method was developed to combine solid-state quantum emitters and spins with integrated photonics at low insertion loss, advancing scalable quantum photonic technologies.

## Graphical abstract

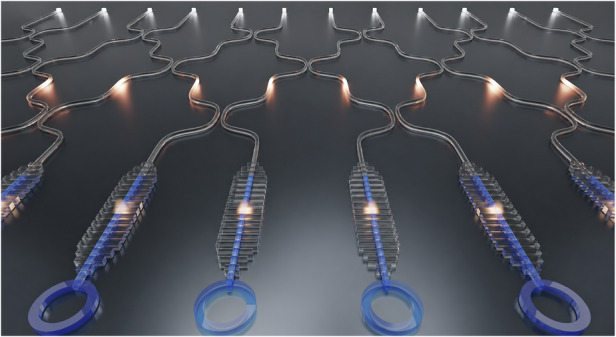

## Introduction

A long-standing goal in quantum photonics is the development of an integrated photonics platform that truly operates in the quantum regime^1,2^. If such a platform can achieve sufficiently high fidelity, efficiency, and scalability, it could enable transformative applications ranging from optical quantum computing^3^ and quantum optical neural networks^4^, to quantum repeaters and quantum networks^5^. What distinguishes quantum integrated photonics from its classical counterpart is its ability to generate, process, and store quantum states of light. Realizing this capability, however, is challenging because it requires strong optical nonlinearities at the single-photon level^6^.

Solid-state quantum emitters, such as semiconductor quantum dots, color centers, and organic molecules, have demonstrated remarkable performance as deterministic sources of single^7–9^ and entangled photons^10^. Moreover, many of these emitters host internal electron and nuclear spin states that can be harnessed for storing photonic quantum states^11^, mediating photon-photon interactions^12,13^, and generating more complex photonic cluster states^14,15^. Integrating such quantum emitters into photonic integrated circuits holds tremendous promise for advancing photonic quantum computing^3,4^ and quantum networking^5^. Despite this potential, progress has been hindered by the disparate and often incompatible material requirements of the individual components. For example, group-IV color centers in diamond are among the leading candidates for realizing spin-photon interfaces^16,17^. However, despite advances in diamond nanofabrication^18–21^, building large-scale photonic circuits directly in diamond remains a grand challenge. Conversely, while materials such as silicon^22^, silicon nitride^23^, and lithium niobate^24^ offer excellent scalability for photonic integration, and exciting progress has been made in identifying quantum emitters in some of these materials^25^, it remains challenging to identify emitters that simultaneously possess large quantum efficiency, large Debye-Waller factor, near transform-limited optical linewidths, and long spin coherence time, as demonstrated by group-IV color centers in diamond.

Heterogeneous integration offers a promising path by combining disparate materials within a single platform, thereby leveraging the advantages of both^26,27^. This approach has been highly successful in classical photonics, for example in integrating III-V lasers with silicon^28^ or other low-loss materials^29,30^. In quantum photonics, however, heterogeneous integration presents unique challenges, as even modest insertion loss at material interfaces can destroy the non-classical features of quantum light. At present, no strategy achieves both low loss and broad material compatibility. Direct embedding of quantum nanoparticles into photonic devices made in another material often introduces substantial scattering and intra-cavity loss^31–34^. Adiabatic mode transfer based on pick-and-place^35–38^, transfer-printing^39,40^, and lock-and-release^41^ techniques typically introduce dB-level insertion loss due to alignment inaccuracies. Top-down heterogeneous integration^42^ improves alignment accuracy, yet its applicability is restricted to host materials that can be fabricated as large membranes and reliably bonded to a heterogeneous substrate, making systems such as diamond particularly challenging. Overcoming these losses and alignment constraints remains essential for realizing scalable quantum photonic integrated circuits.

In this article, we present a heterogeneous quantum photonic integration approach that enables near-unity coupling efficiency at material interfaces through fully self-aligned components. We showcase the method by integrating diamond with titanium dioxide (TiO_2_), an emerging material for integrated photonics at visible wavelengths owing to its wide transparency window, high refractive index, CMOS compatibility, significant Kerr nonlinearity and thermo-optic coefficients, and a modest waveguide propagation loss of 5 dB/cm at 589 nm^43^. Using the resulting devices, we demonstrate Purcell enhancement of a silicon-vacancy (SiV) center in diamond induced by a diamond-TiO_2_ heterogeneous photonic crystal cavity, as well as optical spin control and readout of a SiV center via a TiO_2_ photonic circuit. We further demonstrate that our approach, when combined with inverse photonic design, could enable efficient and broadband collection of single photons from a quantum emitter into a heterogeneous waveguide. Our approach is not limited to color centers in diamond or TiO_2_ photonics. It has the potential to be broadly applicable to a wide range of solid-state quantum emitters integrated with thin-film photonic devices where conformal deposition is feasible. Together, these results represent a significant step toward scalable, high-efficiency integration of solid-state quantum emitters with large-scale photonic circuits.

## Results

### Workflow of the self-aligned heterogeneous photonic integration

**Fig. 1:**
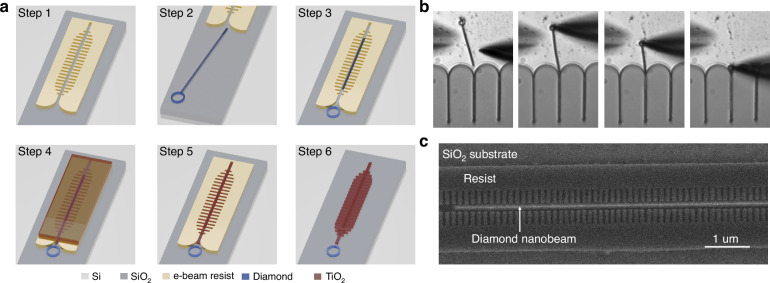
Self-aligned heterogeneous photonic integration. **a** Workflow of the self-aligned heterogeneous photonic integration. Step 1: patterning of the inverse of the full photonic circuit into an e-beam resist layer. Step 2: pick-and-place of a diamond nanobeam near the entrance of the slot defined by the e-beam resist. Step 3: insertion of the diamond nanobeam into the slot via a self-guided process. Step 4: conformal deposition of TiO_2_ over the entire substrate via atomic layer deposition. Step 5: removal of excess TiO_2_ via back-etching. Step 6: removal of the e-beam resist. **b** Optical microscope images showing different stages during the nanobeam insertion process. **c** SEM image of the device right after the insertion of the diamond nanobeam (Step 3)

Figure 1a outlines the workflow of the self-aligned heterogeneous photonic integration. We illustrate the process using a heterogeneous fishbone photonic crystal cavity. However, as we will discuss later, the scheme is general and can be applied to a wide range of photonic geometries. First, we pattern the inverse of the full photonic circuit into an e-beam resist layer on a SiO_2_ substrate (Step 1). A diamond nanobeam is then inserted into its designated slot defined by the e-beam resist (Steps 2 and 3). Following insertion, we conformally deposit TiO_2_ across the chip (Step 4), remove the excess TiO_2_ by back-etching (Step 5), and strip the resist (Step 6). This sequence transfers the resist pattern into TiO_2_ and leaves the diamond nanobeam seamlessly embedded within the TiO_2_ photonic circuit. The Materials and methods section contains details of the heterogeneous device fabrication.

The insertion of the diamond nanobeam is the most critical step in our heterogeneous photonic integration. The diamond nanobeams are fabricated on an electronic-grade single-crystal diamond via electron beam lithography followed by angled etching in a Faraday cage (see Materials and methods). Using a tungsten probe under an optical microscope, we first pick up a nanobeam from the diamond substrate and transfer it to the heterogeneous substrate near the designated slot. We then use the same probe to push the nanobeam into the slot. A ring feature at one end of the nanobeam facilitates engagement between the probe and the beam. To achieve self-alignment, the slot width (230 nm) is designed to be marginally larger than the width of the nanobeam (200 nm). Combined with the large aspect ratio of the nanobeam, this geometry constrains the lateral and angular degrees of freedom and promotes straight insertion. Because the slot and the nanobeam are both subwavelength in width, they are not resolvable under the optical microscope. To address this, the resist pattern incorporates a funnel-shaped entrance that passively guides the nanobeam. As the probe advances the beam, the funnel progressively corrects its orientation, and once engaged, the beam naturally registers with the slot. Figure 1b shows several microscope images captured during the insertion. Figure 1c shows a scanning electron microscopy (SEM) image after successful insertion, which confirms a straight, well-seated nanobeam with no observable damage to the fishbone photonic crystal pattern defined by the resist. Over the course of this work, we attempted to load diamond nanobeams into 31 different device slots, and every single device was successfully populated. In addition, all devices with the proper crack-prevention features survived the subsequent fabrication steps as detailed in Supplementary Section S1. These results demonstrate the exceptional robustness and deterministic nature of our self-aligned integration process.

The primary advantage of our heterogeneous integration approach is that it enables minimal intracavity and out-coupling losses. Because the diamond nanobeam extends across the entire cavity region, the cavity mode is fully supported by the hybrid diamond/TiO_2_ structure. Consequently, the heterogeneous interface introduces very small intracavity loss, as corroborated by the high quality factors obtained from both simulations and experimental measurements, which will be discussed in the following section.

**Fig. 2:**
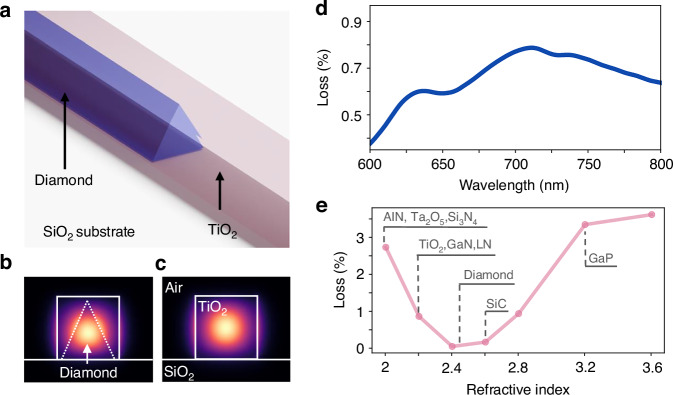
Numerical analysis of external coupling loss for our self-aligned heterogeneous photonic devices. **a** Schematic illustration of the interface at the diamond-TiO_2_ heterogeneous waveguide and a monolithic TiO_2_ waveguide. **b**–**c** Calculated electric-field intensity profile in the cross-section plane of the diamond-TiO_2_ heterogeneous and TiO_2_ monolithic waveguides. **d** Calculated insertion loss at the interface between the two waveguides. **e** Calculated insertion loss at 737 nm as a function of the refractive index of a second material, at the interface between the diamond-in-material heterogeneous waveguide and its monolithic material counterpart

To evaluate the out-coupling efficiency, we numerically analyze the insertion loss at the interface between a heterogeneous diamond/TiO_2_ ridge waveguide and a monolithic TiO_2_ ridge waveguide of identical width, as illustrated in Fig. 2a. Figure 2b–c shows the calculated electric field intensity distributions of the fundamental modes in the heterogeneous and monolithic waveguides, respectively. The two modes exhibit nearly identical spatial profiles and effective refractive indices of 1.89 and 1.79 at 737 nm. Figure 2d shows the simulated insertion loss at the butt-coupled interface. The device achieves an insertion loss of less than 0.8% over a broad wavelength range. While this loss is explored numerically, we provide experimental validation in Supplementary Section S2, where we extract an upper bound for the scattering loss (the loss due to photon scattering into free space) at the interface between the heterogeneous and monolithic waveguides. For the device shown in Fig. 4a, we determine this interfacial scattering loss to be no more than 1.3% at 737 nm.

Another advantage of our integration approach is its potential compatibility with a wide range of material-on-insulator photonic platforms. The diamond nanobeam pick-and-place process relies purely on area-dependent Van der Waals forces between the nanobeam, the tungsten probe, and the SiO_2_ substrate. Because this assembly occurs prior to the deposition of the photonic layer, the process is expected to be largely material-agnostic, provided the photonic layer can be conformally deposited. To explore how refractive index mismatch affects performance in such diverse systems, we calculate in Fig. 2e the insertion loss as a function of the refractive index of the photonic material, at the interface between the diamond-in-material heterogeneous waveguide and its monolithic material counterpart. While the insertion loss increases with a larger refractive index mismatch relative to diamond, it remains below 3.7% across the entire index range from 2 to 3.6. This result suggests that the high spatial overlap of the waveguide modes is the primary factor in maintaining low insertion loss, illustrating the potential for extending our approach to a wide array of other photonic materials. The insertion loss can be further reduced by tapering the diamond nanobeam for adiabatic mode conversion.

### Purcell enhancement by a heterogeneous photonic cavity

**Fig. 3:**
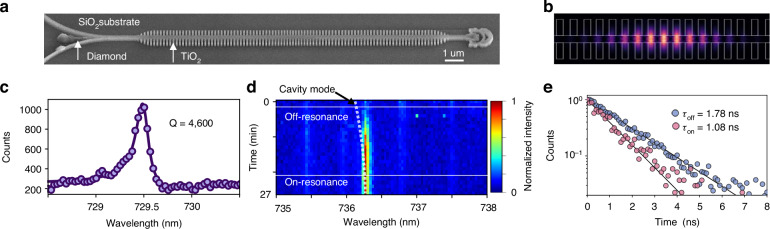
Experimental demonstration of Purcell enhancement induced by a heterogeneous photonic crystal cavity. **a** SEM image of the diamond-TiO_2_ heterogeneous photonic crystal cavity (cavity A). **b** Calculated longitudinal electric field intensity profile of the heterogeneous photonic cavity. **c** Transmission spectrum of a heterogeneous photonic crystal cavity (cavity A), yielding a *Q* factor of 4,600 ± 200. **d** Photoluminescence spectra of the SiV center in a different heterogeneous cavity (cavity B) as the cavity resonance is tuned in-situ via gas condensation. The white dashed line indicates the resonant frequency of the cavity mode. **e** Time-resolved photoluminescence of the same SiV center as in (**d**) when it is on-resonance with (red) and far-detuned from (blue) cavity B

We first demonstrate a fishbone photonic crystal cavity based on our self-aligned heterogeneous integration approach. While high-*Q* heterogeneous cavities utilizing nanodiamonds embedded inside TiO_2_ have been proposed theoretically^44^, experimental demonstrations have remained elusive. Figure 3a shows the SEM image of the heterogeneous diamond-TiO_2_ fishbone photonic crystal cavity (referred to as cavity A). The cavity design is based on a diamond nanobeam with a triangular cross-section embedded inside a TiO_2_ fishbone photonic crystal. Figure 3b shows the simulated electric field intensity of the cavity mode in the propagation plane. The cavity features a field maximum in the center of the diamond nanobeam, crucial for maximizing the coupling with embedded color centers. The cavity resonance is designed to be *λ* = 737 nm, matching the zero-phonon lines of the SiV center. It has a simulated *Q* factor of 120,000 and a mode volume of 1.52${(\frac{\lambda }{n})}^{3}$, where *n* = 2.216 is the refractive index of TiO_2_ at 737 nm.

Figure 3c shows the experimentally measured transmission spectrum of the fishbone photonic crystal cavity (cavity A, see Materials and methods for the experimental setup used throughout this work). The cavity transmission is measured by exciting the cavity center from free space with a super-continuum laser, and collecting the transmitted laser intensity via the inverse-designed grating coupler at the end of the waveguide (see Supplementary Section S3 for the design and characterization of the inverse-designed grating coupler). By fitting the measured spectrum (circles) to a Fano lineshape (solid line), we deduce a cavity linewidth of 0.16 nm and a cavity *Q* factor of 4600 ± 200. In Supplementary Section S4, we provide measured *Q* factors for all heterogeneous photonic crystal cavities fabricated in this work. We consistently observe high-*Q* cavities, which demonstrates the reproducibility of our fabrication and integration approach. Furthermore, a direct comparison with monolithic TiO_2_ photonic crystal cavities fabricated on the same chip indicates that the current *Q* factors are limited by the TiO_2_ nanofabrication process rather than by additional losses introduced by the heterogeneous integration.

To examine the Purcell enhancement induced by the heterogeneous photonic crystal cavity, we utilize a different device (referred to as cavity B) in which a SiV center lies near the cavity center. Cavity B initially had a *Q* factor of 920 with a resonance at ~ 727 nm. Prior to cryogenic measurements, we tuned the cavity resonance close to the SiV zero-phonon lines at 737 nm using repeated atomic layer deposition (ALD) of TiO_2_. This tuning process shifted the resonance as intended, but resulted in a reduced cavity *Q* of 640 (see Supplementary Section S5). Once inside the cryostat, we further tuned the cavity resonance via nitrogen gas condensation, which provides in-situ control of the cavity resonance within a smaller range of 4 nm.

We first measure the cavity-enhanced SiV photoluminescence under a 532-nm continuous wave laser excitation. We excite the SiV center from the free space and collect its emission through the inverse-designed grating coupler at the end of the waveguide. Figure 3d shows the SiV photoluminescence as we tune the wavelength of cavity B. We observe a 6.1 ± 0.6-fold increase in photoluminescence intensity as the cavity is tuned into resonance with the SiV center, suggesting cavity-induced Purcell enhancement.

Complementing the observed intensity enhancement, Fig. 3e shows the time-resolved photoluminescence of the SiV center when it is on resonance with (red circles) and far-detuned from (blue circles) cavity B. For this measurement, a picosecond pulsed laser at 716 nm was used for free-space excitation, with the resulting emission also collected from free space. The lifetime decreases from 1.78 ± 0.10 ns to 1.08 ± 0.07 ns upon reaching resonance, a reduction of ~ 40% that clearly demonstrates the Purcell effect. This lifetime reduction is smaller than the observed intensity enhancement, as expected for SiV centers in diamond which possess significant decay channels not enhanced by the cavity, such as non-radiative decay, phonon-sideband emission, and emission through other three zero-phonon lines that are detuned from the cavity. By assuming an upper bound for the SiV Debye-Waller factor (0.8)^45^ and quantum efficiency (0.3)^46^, and no emission into other zero-phonon lines, we extract a lower bound for the Purcell factor of *F*_*P*_ = 3.7 ± 0.1 (see Supplementary Section S6).

The experimental lower bound of the Purcell factor is approximately 20% of the theoretical maximum (*F*_*P*_ = 21), calculated via ${F}_{P}=\frac{3}{4{\pi }^{2}}\frac{Q}{V}$ while accounting for the minimum possible polarization misalignment between the SiV dipole and the cavity mode. We attribute this discrepancy primarily to the SiV center not being positioned at a field antinode, and to non-enhanced decay channels not fully captured in the calculation of the Purcell factor lower bound. With the maximum cavity *Q* achieved in our experiment (*Q* ≈ 4, 600) and optimized SiV positioning, a Purcell factor of 150 is achievable.

### Chip-integrated optical spin control and readout

**Fig. 4:**
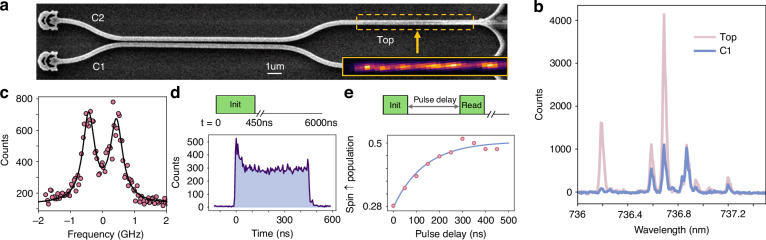
Experimental demonstration of optical spin initialization and readout via a TiO_2_ photonic circuit. **a** SEM image of a TiO_2_ 80/20 insertion coupler integrated with a diamond nanobeam. The diamond nanobeam is embedded inside the upper arm of the TiO_2_ waveguide and the funnel entrance is in the top-right corner. The inset shows the scanning confocal photoluminescence image of the region with the inserted diamond nanobeam, showing a large density of SiV centers embedded inside. **b** Photoluminescence spectra of the SiV centers collected directly from the free space (red) and from the integrated grating coupler at port C1 (blue). **c** Photoluminescence excitation spectrum of the optical C transition of a specific SiV center under a magnetic field around 0.3 T. The two peaks correspond to the two spin-conserving transitions. **d** Optical initialization and readout of the SiV spin via the photonic circuit. **e** Measurement of spin relaxation time via a pump-probe sequence

Besides cavities, our heterogeneous integration approach enables the direct incorporation of diamond color centers into virtually any TiO_2_ photonic circuits. As an example, we demonstrate the integration of diamond SiV centers with a 2 × 2 TiO_2_ insertion coupler. Figure 4a shows the SEM image of the TiO_2_ insertion coupler integrated with a diamond nanobeam from the top right channel. The diamond nanobeam contains a large density of SiV centers, as demonstrated by the scanning photoluminescence image of the region around the inserted diamond nanobeam (inset of Fig. 4a). To demonstrate coupling between the embedded SiV centers and the TiO_2_ photonic circuit, Fig. 4b shows the photoluminescence spectrum when we excite the center of the diamond nanobeam with a 532-nm laser from free space, and collect the signal from either the top (red) and one of the inverse-designed grating couplers following the insertion coupler (blue). The resonance of each emission peak is nearly identical between the two collection channels, confirming that the SiV emission is efficiently coupled into the TiO_2_ photonic circuit. We attribute the difference in the relative intensities of the emission peaks between the two spectra to the polarization-dependent coupling between the SiV emission and the heterogeneous waveguide.

Building on this SiV-integrated TiO_2_ platform, we further demonstrate optical control and readout of a SiV spin through the TiO_2_ photonic circuit. Such chip-integrated optical spin control and readout are essential for multiplexing many quantum memories on a single chip, a key requirement for scalable quantum repeaters and quantum networks^47^. We perform the spin measurement at 5 K with a magnetic field of 0.3 T applied along the diamond [001] crystal axis. Figure 4c shows the photoluminescence excitation spectrum of the optical C transition from this SiV center, revealing two spin-conserving transitions separated by 1 GHz. The linewidths of the two transitions are measured to be 470 ± 20 MHz and 450 ± 20 MHz, respectively. These values are slightly broader than the typical SiV linewidth in our devices. In Supplementary Section S7, we provide a statistical comparison of linewidths and range of spectral diffusion between SiV centers in bulk diamond and those in our heterogeneous devices, demonstrating that the integration process does not degrade the optical coherence properties of the SiV centers.

To demonstrate optical spin control and readout, we inject a resonant laser into the chip via the inverse-designed grating coupler C2 and selectively drive one of the spin-conserving transitions, while collecting the phonon-sideband emission from the coupler C1. The laser power is 0.9 *μ*W, measured before the objective lens. The resonant excitation is modulated by an acousto-optic modulator (AOM) into a periodic 450-ns pulse train with a 6-*μ*s repetition period, as shown in the top panel of Fig. 4d. The bottom panel of Fig. 4d displays the time-resolved histogram of the phonon-sideband signal, which exhibits a clear exponential decay characteristic of optical pumping. These measurements demonstrate that both optical spin initialization and readout can be performed directly through the TiO_2_ photonic circuit.

From Fig. 4d, the fluorescence signal drops to 56% of its initial intensity at steady state. Accounting for the initial thermal distribution (*P*_up_ = *P*_down_ = 0.5), we estimate a final population of 0.28 in the bright state, yielding an initialization fidelity of 0.72. This relatively modest fidelity is likely limited by spin relaxation during the initialization sequence, since the spin relaxation time *T*_1_ is comparable to the optical initialization time. Figure 4e shows a pump-probe measurement of the spin relaxation time *T*_1_, yielding 130 ± 30 ns. From an exponential fit to the optical pumping dynamics, we extract an optical initialization time of 25 ns. The spin initialization fidelity and spin *T*_1_ time are comparable to SiV centers in bulk diamond measured in the same condition (see Supplementary Section S8). We expect that we can improve the spin lifetime by purposely aligning the magnetic field with the SiV symmetry axis^48^, and speed up the optical initialization by driving a spin-non-conserving transition that provides a faster optical decay pathway.

### One-way quantum light emission via inverse design

**Fig. 5:**
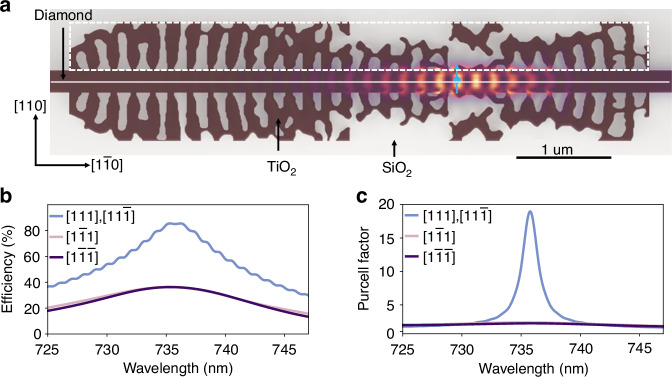
Design and simulation of an inverse-designed one-way quantum light extractor. **a** Design geometry of the quantum light extractor overlaid with the calculated electric field distribution when excited by a dipole (blue arrow) polarized along the TE-mode of the waveguide. Calculated collection efficiency (**b**) and Purcell factor (**c**) for four possible orientations of a SiV center

One unique advantage of our heterogeneous photonic platform is its full compatibility with inverse-designed photonic devices^49^, enabling highly compact and broadband quantum photonic components. This is in stark contrast to heterogeneous integrated photonic circuits based on adiabatic mode conversion. To showcase this capability, we employ inverse photonic design to realize a compact, efficient, and broadband quantum light extractor that collects dipole emission from a single color center into a single waveguide mode propagating only in one direction. Such devices are essential for chip-integrated single-photon sources^7–9^ and reflection-based spin-photon interactions^11–13^. Figure 5a shows the resulting design, overlaid with the electric field distribution when excited by an ideal dipole source polarized along the TE mode of the waveguide. The dipole radiation is efficiently converted into the fundamental TE mode of the heterogeneous waveguide, as indicated by the electric-field mode profile. Note that our heterogeneous photonic platform imposes additional fabrication constraints because the region reserved for diamond nanobeam insertion cannot be etched. To account for this constraint, we define the design region entirely outside the insertion area, as indicated by the white dashed box in Fig. 5a, and we impose mirror symmetry about the waveguide centerline (white solid line). This approach preserves full patterning freedom in the TiO_2_ layer while ensuring that the diamond insertion region remains unetched.

To quantitatively evaluate the performance of the quantum light extractor for a SiV center, we consider a diamond nanobeam fabricated from [001]-cut single-crystal diamond, with the TE mode of the waveguide polarized along the [110] direction. We assume an ideal SiV center whose optical C transition dipole is aligned with its symmetry axis^50^. This symmetry axis, however, has four possible orientations. Figure 5b and c show the calculated collection efficiency and Purcell factor for all four orientations of the SiV center when it is used as the dipole source. For the nearly-TE-polarized orientations ([111] and $[11\bar{1}]$), the device achieves a peak collection efficiency of 84% while simultaneously providing a peak Purcell factor of 18. In contrast, the nearly-TM-polarized orientations ($[1\bar{1}1]$ and $[1\bar{1}\bar{1}]$) exhibit significantly lower collection (~ 30%) and negligible Purcell enhancement, as the device is optimized specifically for TE-polarized emission.

To understand the underlying working principle, we note that the quantum light extractor looks very much like a one-sided cavity, formed by asymmetric Bragg reflectors where the left mirror is designed to be significantly more reflective than the right. Indeed, the resulting peak Purcell factor is consistent with the value calculated based on the simulated Q/V ratio. However, in contrast to conventional photonic crystal cavities, the inverse-designed device maintains a large collection efficiency of above 70% across a 5.5-nm spectral window, which is significantly broader than the cavity linewidth (1.6 nm). This contrast stems from the difference in design strategy. Conventional approaches prioritize a high-*Q* resonance to drive Purcell enhancement, which is inherently narrow-band, and then open one side of the cavity to enable waveguide coupling. In contrast, our inverse-design algorithm optimizes specifically for collection efficiency, treating the photon collection primarily as a mode-matching problem. The device acts as a mode converter that maps the dipole emission pattern into the waveguide mode through multi-mode interference, a process that is fundamentally more broadband. The observed Purcell enhancement is not an imposed design constraint, but rather an emergent property of the optimized geometry required for efficient coupling. The broad bandwidth makes the quantum light extractor particularly suitable for applications in wavelength-multiplexed quantum repeater nodes, where multiple color centers with different wavelengths must be efficiently interfaced on a single chip.

We note that while the inverse-designed quantum light extractor is currently presented as a numerical demonstration, its fabrication feasibility is experimentally supported by the successful implementation of the inverse-designed grating couplers used in Figs. 3 and 4, which showed excellent experimental performance in close agreement with simulations.

## Discussion

The self-aligned heterogeneous integration strategy demonstrated here provides a practical pathway for integrating high-quality solid-state quantum emitters with thin-film photonic technologies. By embedding single-crystal diamond nanobeams directly into TiO_2_ photonic circuits with near-perfect mode matching, our approach significantly reduces the interface losses and alignment errors that have long hindered heterogeneous quantum photonics. This architecture preserves the optical performance of bulk-crystal emitters while leveraging the scalability and design flexibility of thin-film photonic platforms.

In this work, we employ ALD for conformal encapsulation of the inserted diamond nanobeam. Although ALD offers excellent conformality, it is inherently slow and not ideal for producing photonic thin films with thicknesses of several hundred nanometers. However, the underlying integration concept is compatible with other deposition methods that can be engineered to produce conformal thin-film coatings, such as Physical Vapor Deposition (PVD)^51^ or Plasma-Enhanced Chemical Vapor Deposition (PECVD)^52^. Developing conformal deposition processes based on these technologies would provide significantly higher throughput and access to a broader class of photonic materials.

Our cavity *Q* is currently limited by TiO_2_ material loss and nanofabrication precision, rather than the heterogeneous integration. Since the integration process itself is not the limiting factor, our platform has great potential to reach *Q* factors of ~ 20,000 demonstrated in state-of-the-art monolithic TiO_2_ photonic crystal cavities. This would enable a Purcell factor exceeding 600, facilitating high cooperativity for spin-photon interfaces^7–9^, quantum optical nonlinearities^12,13^, and photonic quantum registers^11^. Further improving TiO_2_ nanofabrication could possibly push *Q* factors toward the theoretical limit of 10^5^. Operating in this high-*Q* regime would allow us to precisely quantify the residual intra-cavity losses specifically associated with the heterogeneous integration process. Furthermore, the capability to integrate many quantum emitters within large-scale photonic circuits, combined with inverse-designed photonic devices, makes it possible to realize complex on-chip networks featuring wavelength-selective routing, spatial-mode engineering, and multiplexed spin control and readout, capabilities essential for quantum repeaters based on multiplexed quantum memories^47^.

Beyond circuit-level applications, the heterogeneous photonic architecture naturally supports exploration of photon-mediated collective and many-body interactions by embedding multiple emitters within an engineered photonic environment^53^. Such systems could enable experimental access to dissipative many-body dynamics^54^, long-range correlations^55^, and collective entanglement generation^56^, opening a pathway toward creating synthetic quantum materials based on coupled quantum emitters.

## Materials and methods

### Fabrication of the heterogeneous photonic device

We start the device fabrication by spin-coating a 320-nm-thick layer of ZEP520A onto an HMDS-primed SiO_2_ substrate. The HMDS layer improves the adhesion and mechanical stability of the patterned resist during the subsequent diamond insertion. We then define the photonic structure by exposing the resist with the inverse pattern of the device using an electron-beam writer, followed by resist development. After development, we perform 3 s of O_2_ plasma ashing to remove residual developed resist and exposed HMDS, while leaving the unexposed resist nearly intact. Next, we apply deionized water to the SiO_2_ surface to reduce adhesion between the nanobeam and the substrate, followed by insertion of the diamond nanobeam as described in Fig. 1. After insertion, we perform conformal deposition of TiO_2_ by ALD at 90 °C to a thickness sufficient to fully embed the inserted nanobeam and fill the slot. We then etch back the overgrown TiO_2_ until the resist is re-exposed. We back-etch the TiO_2_ using a chlorine-based ICP-RIE process (300 W ICP power, 150 W RIE power) with gas flows of 12 sccm BCl_3_, 20 sccm Cl_2_, and 8 sccm Ar. Under these conditions, the etch rate is approximately 0.8 nm/s. To maintain precise control over the etch depth, we proceed in multiple etching cycles using 50-nm increments. After each cycle, the sample is removed from the chamber to measure the remaining film thickness using a Filmetrics F50 thin-film measurement system. Once the film thickness falls below 50 nm, we reduce the etching step size to 5 nm to ensure a high precision. Finally, we remove the resist using PG Remover at 70 °C for 20 min, followed by a 3-min Nanostrip clean at 80 °C to remove etch residues, and anneal the device on a hotplate at 250 °C for 2 h to reduce material absorption loss.

### Fabrication of the diamond nanobeam

Diamond fabrication started with silicon ion implantation into electronic-grade diamond at 190 keV with a fluence of 10^12^ ions per cm^2^. High-temperature annealing was then performed at 1150 °C under high vacuum (10^−6^ Torr) to activate optically active defects. Diamond nanobeams were fabricated using an angled-etching technique with a PECVD-deposited silicon nitride hard mask with a thickness of 300 nm. The hard mask was patterned using electron-beam lithography and fluorine-based reactive ion etching to define the nanobeam geometry. Diamond etching was then carried out using an oxygen-based inductively coupled plasma process. The mask pattern was initially transferred into the diamond by a shallow anisotropic etch to establish the nanobeam profile, followed by angled etching using a custom-designed Faraday cage. The Faraday cage redirected incoming ions toward the substrate at oblique angles, enabling controlled undercutting and the formation of fully suspended diamond nanobeams with triangular cross-sections. This geometry was optimized to facilitate subsequent pick-up and transfer processes. After etching, the silicon nitride mask was removed by chemical treatments, yielding clean and well-defined diamond nanobeam structures.

### Measurement setup

We mounted the sample in a closed-cycle cryostat (Montana Instruments) and cooled it to 5 K. Optical excitation and signal collection were carried out using a home-built confocal microscope with a vacuum-compatible objective lens with a numerical aperture of 0.9. For cavity transmission measurements (Fig. 3c), we excited the cavity with a supercontinuum laser with a power of 0.5 mW (measured before the objective lens) and collected the transmitted signal with a spectrometer (Andor). For photoluminescence spectroscopy (Figs. 3d, 4b), we excited the SiV centers with a 532-nm diode laser (Edmund Optics) with a power of 0.4 mW (measured before the objective lens) and collected the emission with the same spectrometer. For time-resolved photoluminescence (Fig. 3e), we excited the SiV center with a picosecond mode-locked laser at 716 nm with a power of 1 mW (measured before the objective lens). For photoluminescence excitation spectroscopy (Fig. 4c), we excited the SiV center near-resonantly with a tunable continuous-wave Ti:sapphire laser (M-Squared) with a power of 0.07 *μ*W (measured before the objective lens) and collected the phonon-sideband emission with a single-photon counting module (Excelitas). For optical spin control and readout (Fig. 4d and e), we mounted the sample on top of a SmCo permanent magnet that applied a 0.3 T field to lift the spin degeneracy. We generated laser pulse sequences by sending the Ti:sapphire output through an acousto-optic modulator (AA Optoelectronic) and time-gating it with a digital signal generator (Swabian Instruments). The laser power is 0.9 *μ*W (measured before the objective lens).

## Supplementary information

Supplementary Information for Self-Aligned Heterogeneous Quantum Photonic Integration

Supplementary Video

## Data Availability

The data generated and/or analyzed are available at 10.5281/zenodo.19836059.
