# Supplementary material for: Self-aligned heterogeneous quantum photonic integration
Source: Light Sci Appl. 2026 Jul 15;15:319. doi: 10.1038/s41377-026-02339-w (PMC13373209; doi:10.1038/s41377-026-02339-w)
Supplement: Supplementary file 1 — Supplementary Information for Self-Aligned Heterogeneous Quantum Photonic Integration [file 41377_2026_2339_MOESM1_ESM.pdf]

Supplementary Information for Self-Aligned Heterogeneous Quantum Photonic Integration

Kinfung Ngan,[1] Yeeun Choi,[2,3] Chun-Chieh Chang,[4] Dongyeon Daniel Kang,[2,5,*] and Shuo Sun[1,*]

[1] *JILA and Department of Physics, University of Colorado Boulder, Colorado 80309, USA*
[2] *Center for Quantum Technology, Korean Institute of Science and Technology (KIST), Seoul 02792, Republic of Korea*
[3] *KU-KIST Graduate School of Converging Science and Technology, Korea University, Seoul, Republic of Korea*
[4] *Center for Integrated Nanotechnologies, Los Alamos National Laboratory, Los Alamos, New Mexico 87545, USA*
[5] *Division of Quantum Information, KIST School, Korea University of Science and Technology (UST), Seoul 02792, Republic of Korea*
(Dated: April 28, 2026)

S1. YIELD OF SELF-ALIGNED HETEROGENEOUS INTEGRATION

The self-aligned heterogeneous integration process consists of two primary stages: the loading of the diamond nanobeam into a lithographically defined slot on the SiO_2 substrate (the "insertion" step) and the subsequent TiO_2 ALD and etch-back (the "filling" step). To provide a comprehensive assessment of the process reliability, we evaluate the yield and robustness of each stage independently.

1. The Insertion Step

The integration process begins with the deterministic insertion of a diamond nanobeam into a lithographically defined slot on the host SiO_2 substrate. To facilitate this, the slot entrance incorporates a funnel-shaped geometry that provides a high tolerance for initial angular and lateral misalignments. As the tungsten probe advances the nanobeam, the funnel progressively corrects its orientation, allowing the beam to naturally register and self-align within the slot.

The accompanying Supplementary Video provides a real-time recording of the process, beginning from the moment the diamond nanobeam is placed on the SiO_2 substrate near the funnel entrance. As demonstrated in the video, the entire insertion is extremely straightforward and is typically completed within 20 seconds, requiring minimal manual adjustment due to the passive guiding of the funnel.

The insertion step is remarkably robust. Throughout our work, we targeted a total of 31 device slots, including 23 photonic crystal cavities (similar to Fig. 3a of the main text) and 8 insertion couplers (similar to Fig. 4a of the main text). These devices were fabricated across 4 different chips in 4 separate fabrication runs. Every single one of these 31 attempts was successful. This represents a perfect 100% loading yield, demonstrating the high reliability and deterministic nature of our integration approach. Furthermore, we successfully loaded a curved diamond nanobeam (with a radius of curvature of 100 μm) into a straight slot defined by the e-beam resist (Fig. S3a). The ability to accommodate such geometric discrepancies further underscores the mechanical robustness and alignment tolerance inherent to our insertion process.

While the loading yield is 100%, in rare instances the loading requires more than one diamond nanobeam to successfully populate a single slot. This happens when a nanobeam is initially dropped too far from the funnel entrance or with a significant initial misalignment. Under these conditions, we have to reposition the nanobeam on the SiO_2 substrate using the tungsten probe, during which the mechanical stress can cause the nanobeam to bend and eventually fracture. Fortunately, because this micromanipulation occurs while the diamond nanobeam is sitting on the bare SiO_2 substrate, the device slot itself remains undamaged. Therefore, any fractured nanobeam can simply be removed and the pick-and-place procedure can be repeated with a new nanobeam until the slot is successfully loaded. Throughout our experiment, a total of 34 diamond nanobeams were consumed to complete the 31 targeted devices.

2. The Filling Step

Once the device slot is successfully populated with a diamond nanobeam, the "filling" process begins with the conformal deposition of TiO_2 via atomic layer deposition (ALD), followed by overgrowth, back-etching, and finally

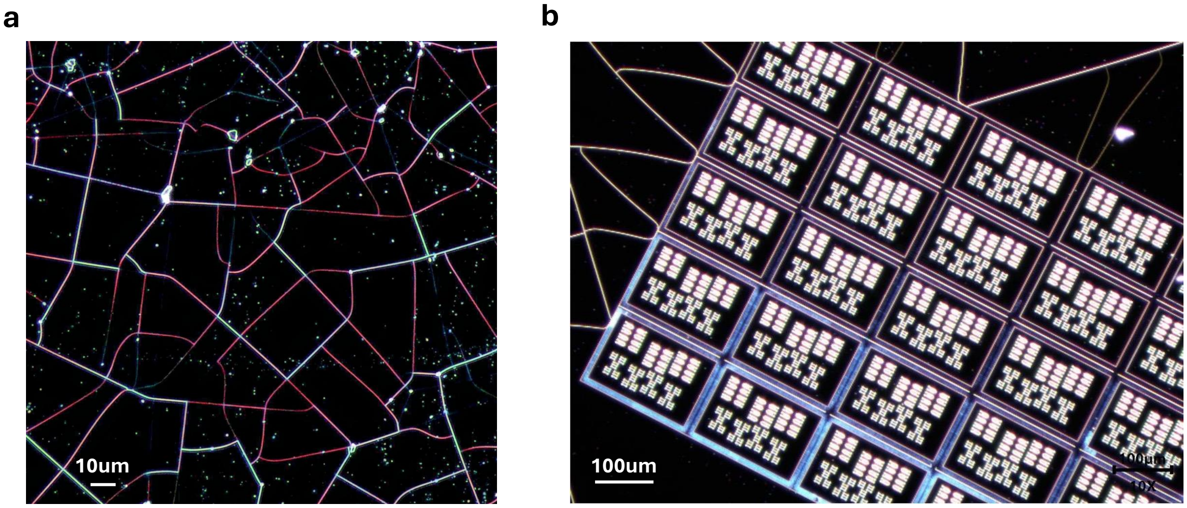

Fig. S1. Optical microscope images of the TiO_2 thin film following overgrowth, in the absence (a) and presence (b) of the crack-stop boundaries.

the removal of the e-beam resist.

The primary failure mechanism identified during initial fabrication runs was the cracking of the TiO_2 thin film. As the TiO_2 overgrowth reaches a thickness of approximately 400 nm, it introduces significant internal stress. Because we utilize a positive e-beam resist (ZEP520A), the majority of the chip remains covered by the resist after development, meaning most of the TiO_2 film sits on a soft polymer layer. The resist is unable to withstand the accumulated stress, and the resulting lateral deformation leads to crack formation. These cracks can propagate across the entire film, causing catastrophic damage to all devices, independent of whether a diamond nanobeam is integrated or not. Figure S1a shows an optical microscope image of the cracked thin film following the TiO_2 overgrowth.

To address this issue, we modified the lithographic pattern to remove a large area of e-beam resist surrounding the devices. These exposed areas allow the deposited TiO_2 film to bond directly to the underlying SiO_2 substrate, providing the mechanical anchoring necessary to suppress crack propagation. Figure S1b shows the optical microscope image of the TiO_2 thin film with these "crack-stop" boundaries. The removal of the resist in these boundary areas successfully prevents cracks from propagating into the device region.

The effectiveness of this technique is reflected in our fabrication yield. Of the 31 devices with successful diamond nanobeam loading, 12 were processed in the initial run before the implementation of crack-stop boundaries, and all 12 were lost to cracking. Following the introduction of the crack-stop boundaries, all 19 remaining devices successfully survived the ALD deposition, back-etching, and resist stripping processes, representing a 100% yield for the filling step.

One drawback of defining the crack-stop boundaries is the significantly increased e-beam exposure time. Since a positive e-beam resist is used, creating the crack-stop boundaries requires electron-beam exposure over large areas surrounding the device regions. This requirement has practically limited the number of devices per run and the total number of fabrication runs we can have in this work. One solution to this throughput bottleneck is to use a negative e-beam resist (such as the AR-N or HSQ). This would substantially reduce the lithography time while maintaining the optimal mechanical support necessary to suppress film cracking.

S2. EXPERIMENTAL MEASUREMENT OF INSERTION LOSS

In Fig. 2 of the main text, we present numerical simulations of the insertion loss at the interface between the heterogeneous diamond/TiO_2 waveguide and the monolithic TiO_2 waveguide. The results indicate that the insertion loss at this interface remains below 0.8% over a broad wavelength range. Experimentally characterizing this insertion loss is challenging due to our specific device geometry. In a standard photonic circuit, loss is typically measured via transmission between two calibrated ports. However, in our architecture, the funnel entrance physically occupies

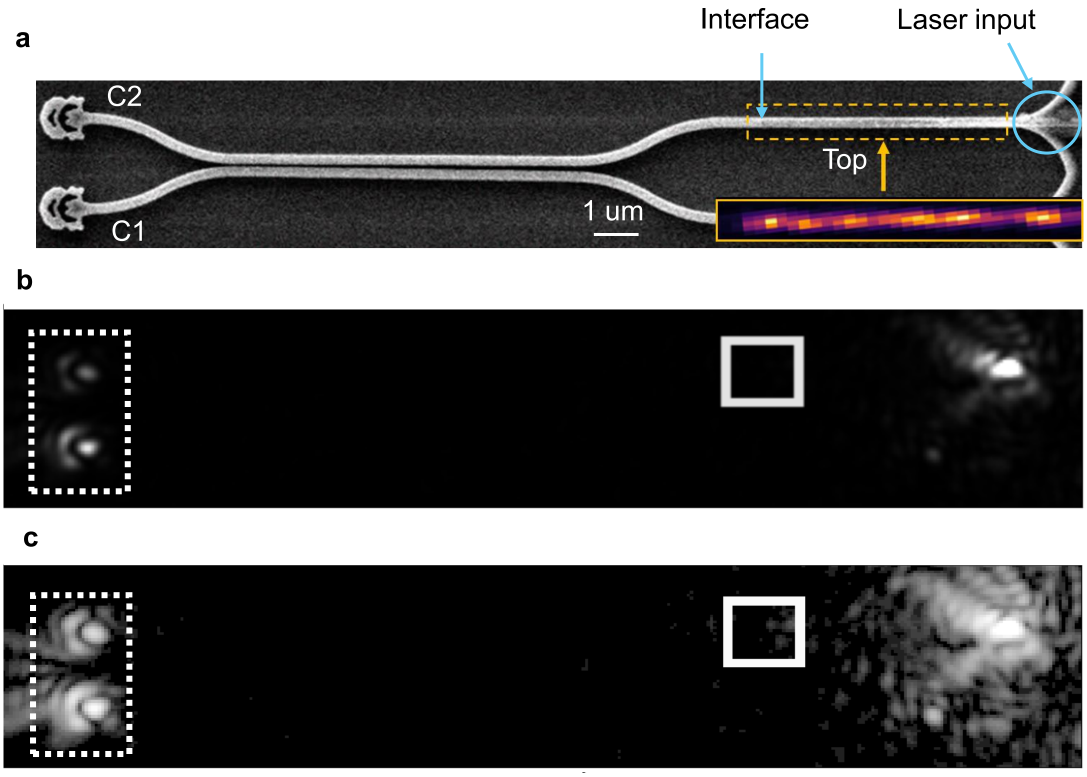

Fig. S2. Characterization of scattering loss at the interface between the heterogeneous and monolithic waveguides. (a) SEM image of the heterogeneously integrated insertion coupler (reproduced from Fig. 4a of the main text). The blue arrow marks the position of the interface between the monolithic and heterogeneous waveguides. The blue circle marks the position of the laser excitation in the experiment shown in (b) and (c). (b) Optical microscope image under laser illumination at the funnel entrance. While strong scattering is visible at the excitation and the two output grating couplers, no noticeable scattering is observed at the waveguide interface on a linear intensity scale. (c) Logarithmic intensity plot of the image in (b). The white solid box indicates the conservatively oversized region used to integrate the interfacial scattering signal, while the dashed box indicates the area used to integrate the grating coupler emission.

the space where an input grating coupler would normally be located. This prevents a conventional through-port transmission measurement.

Nevertheless, since any discrepancy between the physical device and the numerical model would primarily arise from the scattering of photons into free space (due to factors such as misalignment or surface roughness), we can infer the additional insertion loss not accounted for by the simulation by directly monitoring the scattering at the interface. Figure S2a shows a scanning electron microscope (SEM) image of the heterogeneously integrated insertion coupler (same as Fig. 4a of the main text). To measure the scattering loss at the waveguide interface, we mounted the device in our confocal microscope setup and excited the funnel entrance with a continuous-wave laser at 737 nm with a power $\sim$50 μW, measured before the objective. The funnel entrance couples the laser light into the heterogeneous waveguide, which then propagates through the insertion coupler and leads to emission from the two output grating couplers, C1 and C2.

Figure S2b shows the optical microscope image of the device in the presence of the laser illumination. We clearly observe strong scattering at the funnel entrance due to the laser illumination, along with emission from the two grating couplers. Notably, no noticeable scattered light is observed at the location of the interface. This position can be precisely identified by the scanning confocal photoluminescence image shown in the inset of Fig. S2a. The sharp transition in the photoluminescence intensity marks the interface, as SiV centers are present only within the diamond nanobeam.

To quantify the scattering losses, Fig. S2c displays the same optical image using a logarithmic intensity scale. Under these conditions, we observe a faint signal within a deliberately oversized region encompassing the interface (white solid box, Fig. S2c). By integrating the intensity over this generous area to ensure a conservative upper bound and comparing it to the total intensity emitted by the two grating couplers (integrated over the white dashed box), we

determine that the total intensity of the scattered light detected by the camera, $I_{scatter}$, is at most 0.95% of the total emission from the grating couplers detected by the camera, I_{gc}.

We define the scattering loss as

$$\eta_{scatter} = P_{scatter}/P_0,$$

where P_0 is the total laser power inside the heterogeneous waveguide and $P_{scatter}$ is the total power scattered at the interface into free space. The total count of interface scattering detected by the camera, $I_{scatter}$, relates to the total scattering power $P_{scatter}$ by:

$$I_{scatter} = \eta_{camera} \cdot \eta_{obj} \cdot P_{scatter}$$

where η_{camera} is the quantum efficiency of the camera and η_{obj} is the fraction of scattered light collected by the objective lens. Similarly, the total count of grating coupler emission detected by the camera, I_{gc}, relates to the total power in the heterogeneous waveguide P_0 by:

$$I_{gc} = \eta_{camera} \cdot \eta_{gc} \cdot \eta_{bs} \cdot (P_0 - P_{scatter}),$$

where η_{gc} is the efficiency of the grating coupler and η_{bs} is the transmission efficiency of the insertion coupler (i.e., the ratio of total power in the output waveguides to the power in the input waveguides). Combining these equations, we find the scattering loss at the interface to be:

$$\eta_{scatter} = \frac{I_{scatter}}{I_{scatter} + I_{gc} \cdot \frac{\eta_{obj}}{\eta_{gc} \cdot \eta_{bs}}}.$$

To calculate the upper bound of the scattering loss $\eta_{scatter}$, we use the measured upper bound ratio $I_{scatter}/I_{gc}$ of 0.95%, the upper bound value of η_{gc} of 38% obtained from the simulated grating coupler efficiency (Fig. S5c), and the upper bound value of η_{bs} of 1. To determine η_{obj}, we assume isotropic scattering over a 4π solid angle. Given the numerical aperture of 0.9 of our objective lens, we calculate a collection efficiency of $\eta_{obj} = 28\%$. Using these values, we determine the upper bound for the scattering loss at the interface to be $\eta_{scatter} = 1.3\%$.

Throughout this work, we fabricated 4 insertion couplers in total. Two did not function as efficient couplers due to a design error. The remaining two were successful, with one presented in Fig. S2. For the other functional insertion coupler, we successfully integrated a curved diamond nanobeam with a radius of curvature of 100 μm (Fig. S3a). Figure S3b shows the optical microscope image of the device under laser illumination at the funnel entrance. We again observed strong emission from the two grating couplers, with nearly no detectable scattering at the interface between the heterogeneous and monolithic waveguides.

Figure S3c displays the same optical image using a logarithmic intensity scale. By following the same analysis used for the straight device, we determined the upper bound of the scattering loss at the interface to be 7.4%. This scattering loss is slightly higher than that of the straight-nanobeam device, likely due to a minor angular mismatch at the interface and localized mode distortion caused by the curvature of the nanobeam. Nevertheless, these results demonstrate that we can reliably achieve low insertion loss across multiple devices and complex geometries.

Further experimental evidence of low insertion loss at the interface is provided by the relative photoluminescence intensities of the SiV centers. Figure S4 shows the PL spectra of several SiV centers located at various positions within the heterogeneous waveguide shown in Fig. S2. In these measurements, we excited the SiV centers from free space using a green laser ($\sim$0.4 mW, measured before the objective) through the confocal microscope and collected the resulting emission either directly from free space via the confocal microscope (red) or through the output grating coupler, C1 (blue). Despite the fact that the signal must propagate through the interface between the monolithic and the heterogeneous waveguide as well as the subsequent 2-by-2 insertion coupler (beam splitter), we routinely observe emission peaks that are stronger when collected via the grating coupler than directly from free space. This observation confirms that the photons coupled into the heterogeneous waveguide are efficiently guided through the interface into the monolithic waveguide with minimal insertion loss. Notably, not all emission peaks are expected to be stronger at the grating coupler, as the coupling between a specific transition and the waveguide is highly sensitive to the position and dipole polarization of the SiV center.

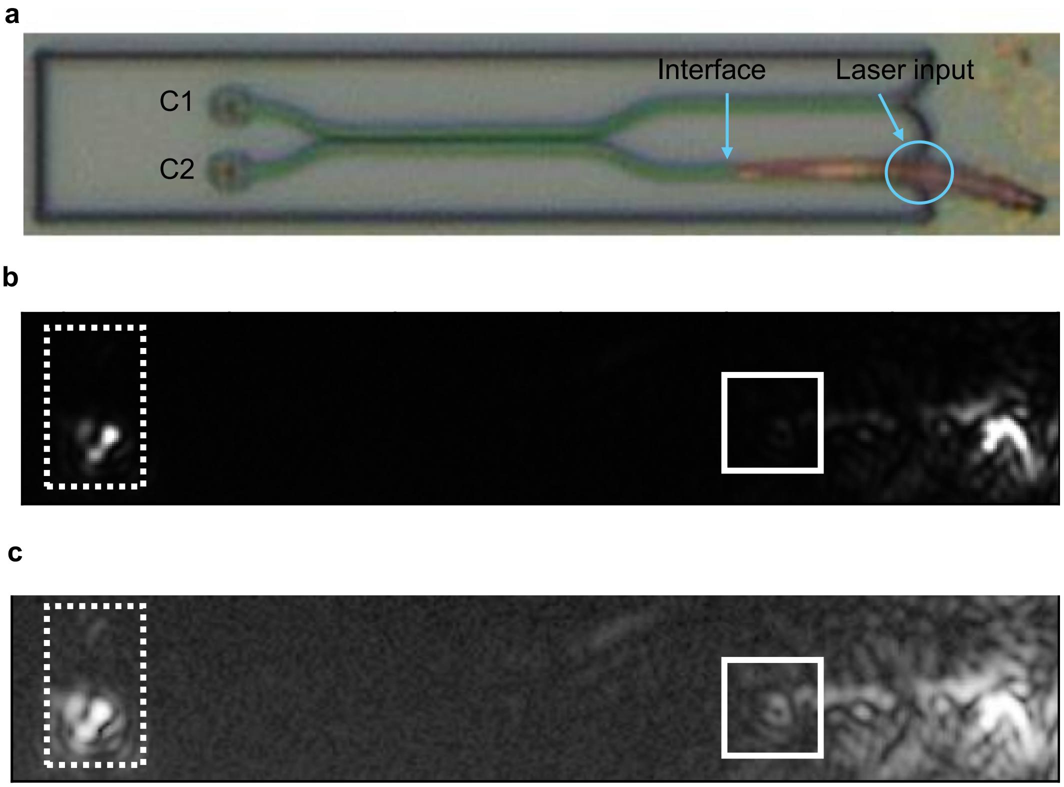

Fig. S3. Characterization of the interfacial scattering loss for a second device integrated with a curved diamond nanobeam. (a) Optical microscope image of the heterogeneously integrated insertion coupler. A curved diamond nanobeam with a radius of curvature of 100 μm is successfully integrated into a straight slot, demonstrating the mechanical robustness and geometric tolerance of the loading process. (b) Optical microscope image under laser illumination at the funnel entrance. Similar to the straight-nanobeam device, strong emission is observed at the output grating couplers with no detectable scattering at the waveguide interface on a linear scale. (c) Logarithmic intensity plot of the image in (b). The white solid box indicates the deliberately oversized region encompassing the interface, while the white dashed box indicates the area used to integrate the grating coupler emission.

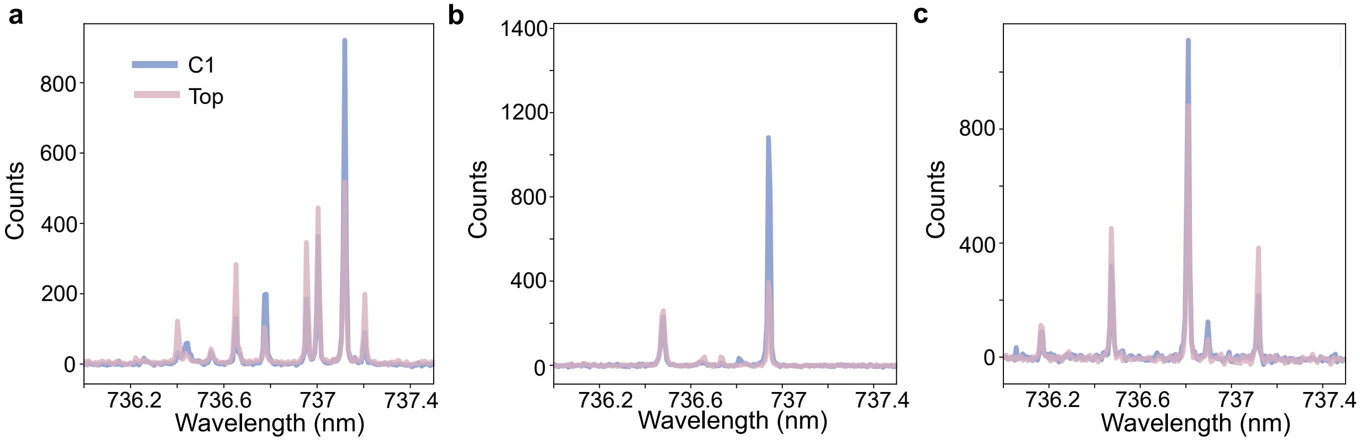

Fig. S4. Photoluminescence spectra from multiple SiV centers located at three different positions (a, b, c) within the heterogeneous waveguide. For each emitter, the signal was collected either directly from free space via the confocal microscope (red) or through the output grating coupler C1 (blue). Despite the additional path loss from the interface, the insertion coupler, and the grating coupler, we routinely observe emission peaks that are stronger when collected via the output grating coupler. This contrast verifies that the insertion loss at the interface between the heterogeneous and monolithic waveguides is minimal.

S3. INVERSE-DESIGNED GRATING COUPLER

The grating coupler employed in this work is not a fundamental requirement of our heterogeneous integration platform. Rather, it serves as a facilitatory component to enable all measurements, including color center spectroscopy, waveguide/cavity transmission, and optical spin control and readout, to be performed via the same confocal microscope setup. This approach allows us to bypass the experimental complexity and mechanical overhead of building a cryogenic chip-to-fiber interface.

For this purpose, we prioritized a solution that could simultaneously satisfy three key design and fabrication constraints:

1) Fabrication Simplicity: The coupler must be realized in a single full-etch step, avoiding the shallow etching step typically required to break vertical symmetry in high-efficiency grating couplers [1].

2) Vertical Collection: A perfectly vertical collection angle (0°) was required to simplify the alignment of the confocal microscope setup.

3) Broad Bandwidth: A large transmission bandwidth was necessary to ensure reliable coupling despite variations in resonant wavelengths across different cavities.

Given the difficulty of satisfying these combined constraints using a conventional periodic design, particularly the requirement for high-efficiency vertical collection without shallow etching, we employed inverse photonic design. This approach has been successfully demonstrated by some of our authors in the past to create efficient vertical grating couplers in monolithic diamond photonics [2].

Figure S5a shows the design of the vertical grating coupler used in this work. The inverse-designed structure was obtained via adjoint-based topology optimization implemented with Tidy3D (Flexcompute) [3]. The coupler has a footprint of $1.4\times1.6\ \mu\text{m}^2$ and is optimized to couple the fundamental waveguide mode into a vertically emitted Gaussian beam with a beam waist diameter of 0.52 μm. The blue solid line in Fig. S5c shows the calculated coupling efficiency as a function of wavelength. The design achieves a peak efficiency of 38%. Notably, although we did not apply multi-frequency optimization, the design naturally exhibits a broad coupling bandwidth, maintaining an efficiency exceeding 30% over a window of more than 70 nm.

Figure S5b shows the SEM image of the inverse-designed grating coupler. To characterize the coupling efficiency, we fabricated a waveguide terminated by identical couplers at both ends. Figure S5d shows a confocal optical microscope image of this waveguide. When we excite the input grating coupler with a continuous-wave laser, we observe a strong Gaussian-like emission from the output coupler, indicating efficient coupling. The red solid line in Fig. S5c shows the experimentally measured coupling efficiency of a single grating coupler as the laser wavelength is tuned. We achieve a peak efficiency of 18% with a full width at half maximum (FWHM) of 40 nm. We note that this value is a lower bound because our calibration assumes perfect coupling between the Gaussian mode and the fiber, and neglects waveguide propagation loss. If we assume a reasonable fiber coupling efficiency of 50%, then the peak efficiency of the grating coupler would be 36%, which agrees well with the simulated peak value of 38%.

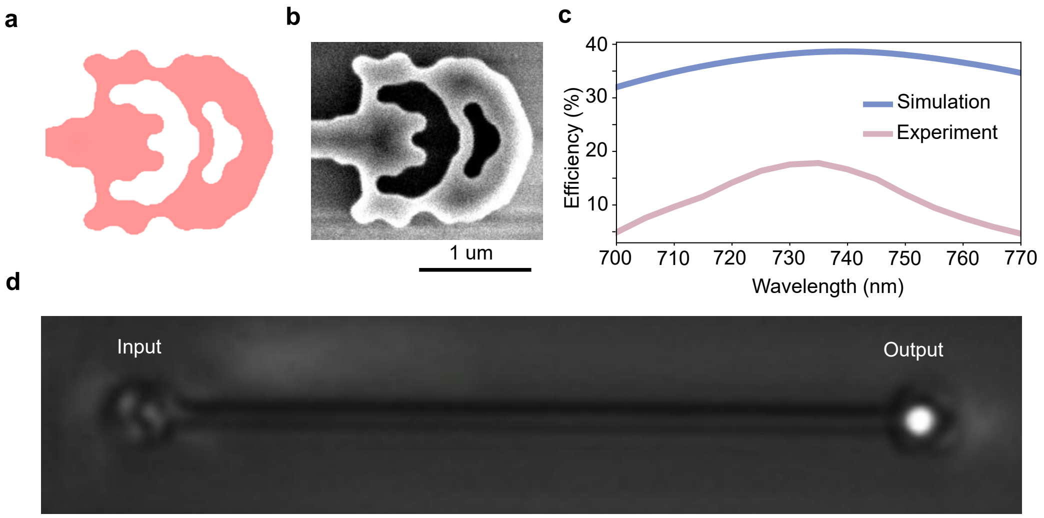

Fig. S5. Design and characterization of the inverse-designed grating coupler. (a) Design of the vertical grating coupler. (b) SEM image of the fabricated grating coupler. (c) The coupling efficiency of the grating coupler as a function of wavelength. Blue solid line shows the calculated values, and red solid line shows the experimentally measured values. (d) Optical microscope image of a waveguide when excited by a laser at the input grating coupler. A clear Gaussian-like emission at the output is visible, indicating good coupling efficiency from the Gaussian beam to the waveguide fundamental mode.

S4. LIMITATIONS OF THE CAVITY QUALITY FACTOR

In the main text, we present measurement results for two heterogeneously integrated photonic crystal cavities, referred to as cavity A and cavity B. Over the course of this work, we fabricated a total of 23 photonic crystal cavities in 4 different chips across 4 fabrication runs. Out of the 23 cavities, 8 of them were processed in the initial run prior to the development of our crack-prevention approach and were consequently lost to film cracking. The transmission spectra, and measured Q factors for the remaining 15 photonic crystal devices are shown in Fig. S6. Of these 15 devices, 9 were fabricated in the first run after we developed the crack-prevention approach (Fig. S6a–i, including cavity A shown in Fig. S6b), 2 in the second run (Fig. S6j–k), and 4 in the final run (Fig. S6l–o, including cavity B shown in Fig. S6n).

As shown in Fig. S6, we consistently produced high-Q cavities in the first fabrication run, with 6 of the 9 devices achieving a Q factor exceeding 2,000. For all 9 cavities from this first run, we extracted a mean Q of 2,500 with a standard deviation of 930. In contrast, the second and third fabrication runs yielded significantly lower cavity Q factors (including the cavity B we used for the Purcell enhancement demonstration in Fig. 3d and 3e of the main text), which we attribute to the declining performance of the electron-beam writer. Throughout the duration of this project, the stability of the electron-beam writer degraded significantly. In fact, the system was decommissioned for a full replacement immediately following our final fabrication run. This hardware instability resulted in less precise feature definition and increased geometric deviations in the later runs, ultimately limiting the achievable Q factors.

An important question is whether the cavity Q is limited by losses introduced by the heterogeneous integration. To investigate this, we measured the cavity Q of monolithic TiO_2 photonic crystal cavities fabricated on the same chip during the first run. These devices underwent the same fabrication process, with the sole exception that no diamond nanobeams were loaded into the defined slots prior to the TiO_2 deposition. Figure S7a shows the simulated mode profile of the monolithic photonic crystal cavity. The mode profile is very similar to that of the heterogeneous cavity (Fig. S7b, same as Fig. 3b of the main text), which is expected given the similar refractive indices of TiO_2 and diamond. The monolithic cavity has a simulated resonance of 706 nm, blue-shifted from the heterogeneous cavity (737 nm) due to the slightly lower refractive index of TiO_2, and a theoretical Q of 140,000.

Figure S8 shows the measured transmission spectra and Q factors for 10 randomly selected monolithic photonic crystal cavities, from which we extracted a mean Q of 1,020 and a standard deviation of 230. Notably, the measured Q of these monolithic devices is significantly lower than that of the heterogeneous cavities. This leads to two key conclusions. First, any loss introduced by the heterogeneous integration is not a primary factor limiting the performance of our current heterogeneous devices. Rather, the cavity Q is limited by the TiO_2 nanofabrication itself. Second, the

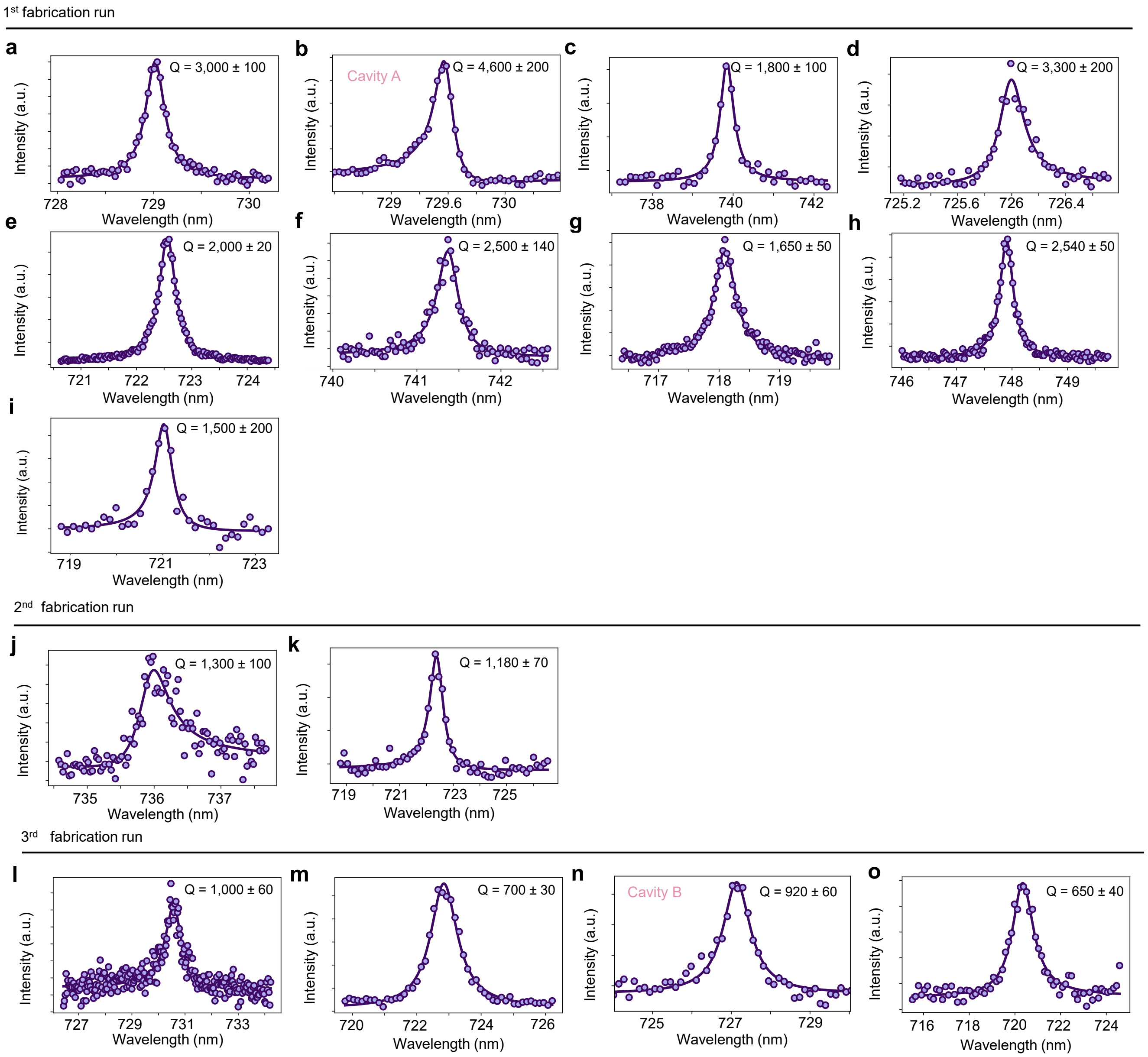

Fig. S6. Transmission spectra and measured Q factors of a total of 15 heterogeneously integrated photonic crystal cavities fabricated in this work. Devices shown in panel a - i are fabricated on the same chip in the first run after we developed the crack-prevention technique, j - k are on the same chip in the second run, and l - o are on the same chip in the final run.

fact that the heterogeneously integrated cavities exhibit higher Q suggests that considerable absorption or scattering exists within the TiO_2 material. By replacing a portion of the TiO_2 with high-quality single-crystal diamond, the modal overlap with the lossy TiO_2 is reduced, thereby increasing the Q factor. We note that the scattering or absorption inside the TiO_2 material is not a fundamental limit, as monolithic ALD-based TiO_2 photonic crystal cavities with Q factors of 20,000 have been demonstrated [4]. Future work on refining the ALD and annealing parameters is therefore expected to improve the Q factor of both the monolithic and heterogeneous cavities.

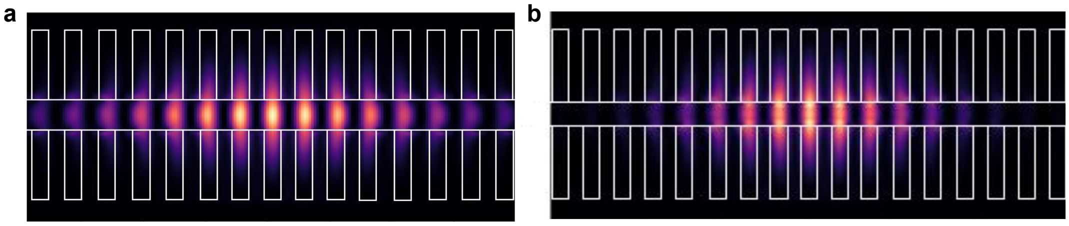

Fig. S7. Calculated longitudinal electric field intensity profile of the monolithic photonic crystal cavity (a) and the heterogeneous photonic crystal cavity (b). Panel (b) is identical to Fig. 3b of the main text.

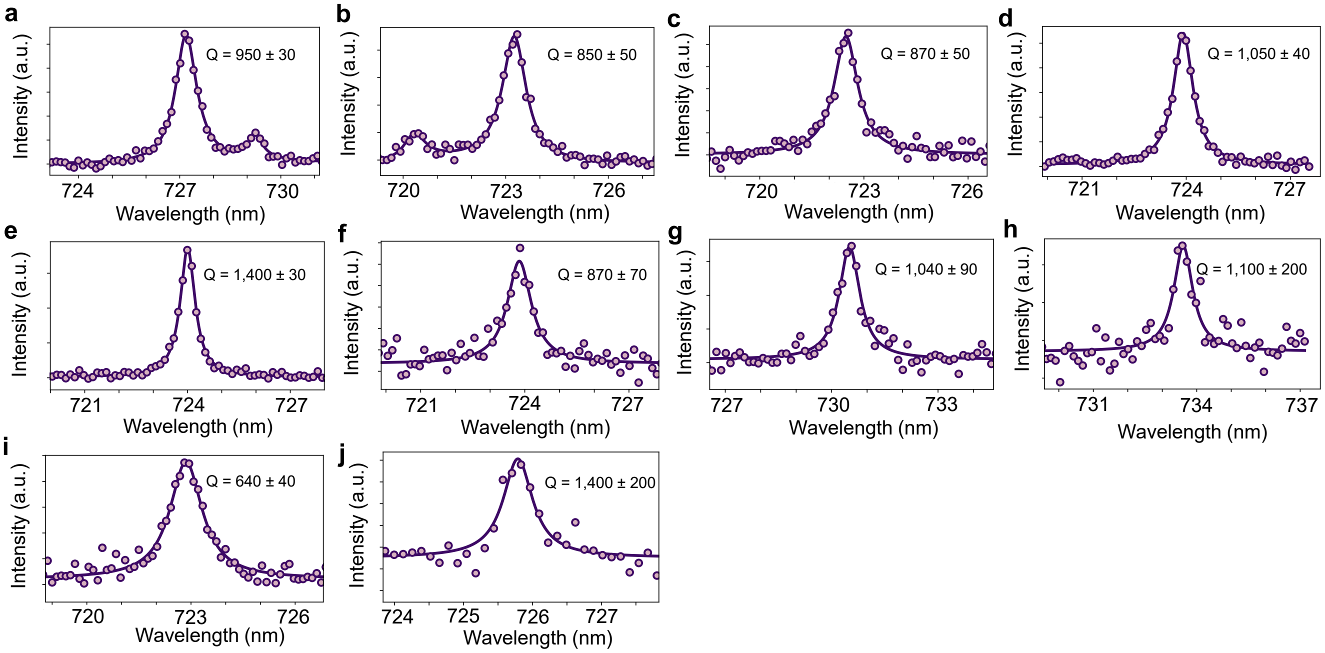

Fig. S8. Transmission spectra and measured Q factors for 10 randomly selected monolithic TiO_2 photonic crystal cavities in the first fab run after we developed the crack-prevention technique.

Finally, we study how the electron beam lithography precision limits the cavity Q. Figure S9a shows the SEM image of the heterogeneously integrated "cavity A" reported in the main text, which has a measured Q factor of 4,600. Figure S9b shows the cavity geometry extracted from the SEM image. We observe two major deviations between the extracted cavity geometry and the original design. First, while the original design specifies each fin of the photonic crystal as a perfect rectangle, the fabricated device exhibits rounded corners with a radius of curvature (ROC) of ∼60 nm. Second, the lateral dimensions of the structures, including both the waveguide width and the average fin width, are ∼10% larger than the intended design values. This expansion is likely a result of overexposure during the electron-beam lithography process. The calculated Q factor of the extracted cavity geometry is 5,200, with a resonance near 730 nm, in good agreement with our experimental measurement. This suggests that besides the loss introduced by the TiO_2 material, the current precision of the electron beam lithography also limits the cavity Q factor.

To understand the pathways for improving the cavity Q, we simulated the Q factor as a function of the fin corner ROC and the fractional lateral dimension deviation (encompassing both the waveguide and fin widths). Figure S9c illustrates the calculated Q as a function of these two parameters. Our simulations indicate that the cavity Q could be improved to 10^4 by reducing the fin corner ROC from 60 nm to 40 nm, or by decreasing the fractional dimension deviation from 10% to 5%. Reaching a Q factor of 10^5 would require further refining the dimension deviation to below 5% and the fin corner ROC to below 30 nm. Such precision is within the capabilities of state-of-the-art electron-beam lithography systems. Once the measured cavity Q is no longer dominated by lithographic errors, we will be able to more accurately assess any secondary limitations imposed by the heterogeneous integration process.

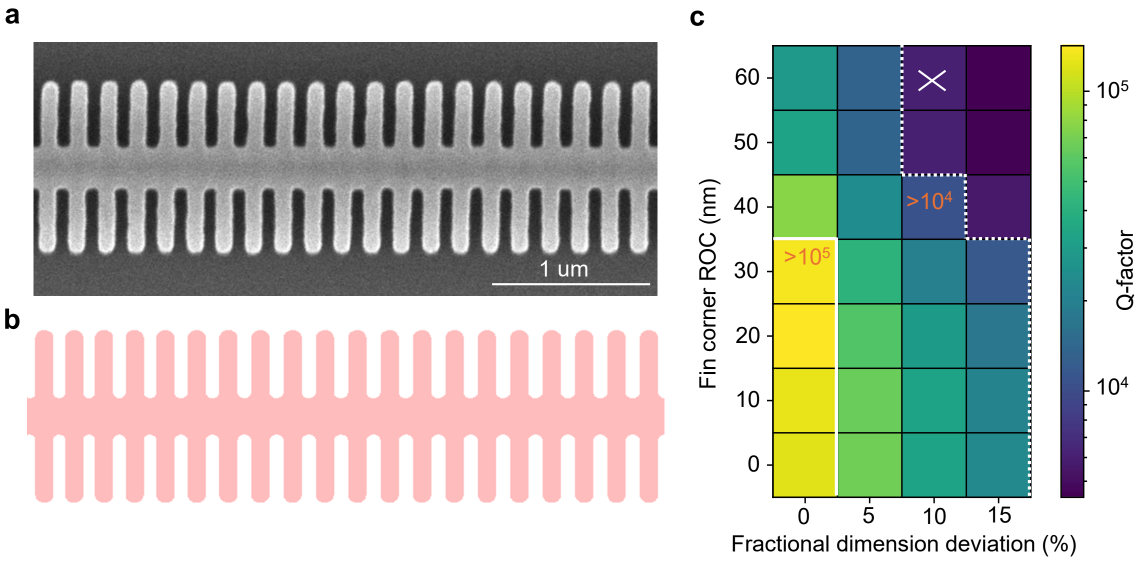

Fig. S9. (a) SEM image of the fabricated heterogeneous photonic crystal cavity shown in Fig. 3a of the main text, featuring a cavity Q of 4,600. (b) The extracted pattern of the cavity based on (a). (c) Simulated cavity Q as a function of fin corner ROC and the fractional lateral dimension deviation. The X sign indicates the level of geometry deviation of our current device.

S5. CHARACTERIZATION AND TUNING OF THE CAVITY USED FOR PURCELL ENHANCEMENT

Figure S10a shows the spectrum of the photonic crystal cavity used for the Purcell-enhancement experiment (cavity B) . The cavity resonance is initially at ∼727 nm, which is blue-detuned from the SiV zero-phonon line by 10 nm. This detuning exceeds the in-situ tuning range achievable via gas condensation (∼4 nm). We therefore explored an alternative strategy for cavity wavelength tuning. By depositing TiO_2 on top of the device via ALD, we can red shift the cavity resonance, and the exact amount of shift depends on the ALD layer thickness. Figure S10b shows the measured cavity spectrum after 20 ALD cycles. We observe a shift of ∼ 2 nm and, at the same time, a reduction in cavity Q from 920 to 860, which we attribute to a mismatch in cavity thickness between the design and the post-deposition device. Figure S10c shows the measured cavity spectrum after 120 ALD cycles. We successfully shift the cavity resonance to 737 nm. However, the cavity Q decreases further to 640. This reduced Q limits the Purcell factor achievable with this device.

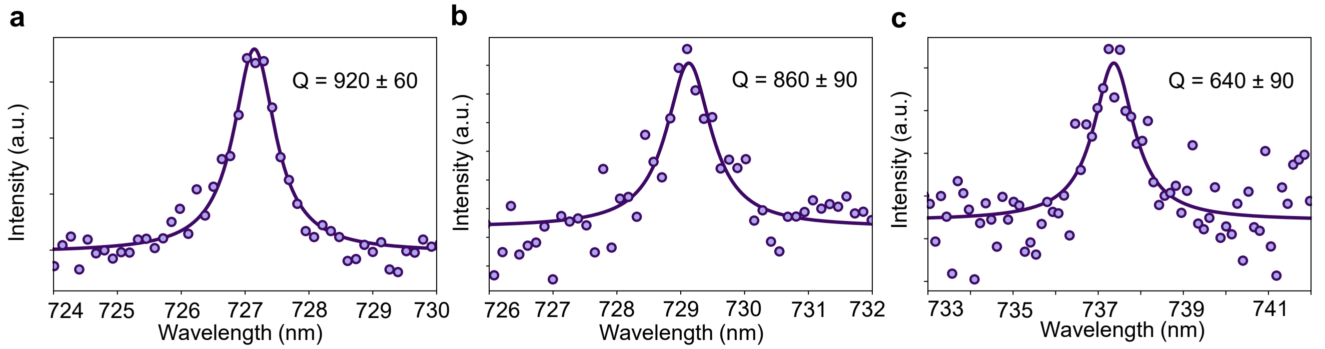

Fig. S10. Transmission spectrum of cavity B used for the Purcell enhancement experiment (Fig. 3d and 3e of the main text) right after the device fabrication (a), after 20 ALD deposition cycles (b), and after a total of 120 ALD deposition cycles (c). The cavity spectrum was measured from a weak and broadband TiO_2 autofluorescence that is enhanced at the cavity resonance. In all panels, the circles are the measured data, and the solid line is a fit to a Lorentzian function from which we extract the cavity Q factor.

S6. CALCULATION OF THE LOWER BOUND OF THE PURCELL FACTOR

Fig. 3e of the main text shows the time-resolved photoluminescence of the SiV center when it is on and off resonance with the cavity mode, from which we extract $\tau_{\text{on}} = 1.08 \pm 0.07$ ns and $\tau_{\text{off}} = 1.78 \pm 0.1$ ns. For an ideal two-level system coupled to an optical cavity, the Purcell factor F can be directly inferred from the ratio of these two lifetimes: $F = \tau_{\text{off}}/\tau_{\text{on}}$. However, for the SiV center, several decay channels are not enhanced by the cavity, including non-radiative decay, phonon sideband emission, and decay through other zero-phonon lines that are far detuned from the cavity resonance. Therefore, τ_{on} and τ_{off} are related by

$$\frac{1}{\tau_{\text{on}}} = (\xi F + (1 - \xi)) \frac{1}{\tau_{\text{off}}},$$

where ξ is the fraction of decay occurring through spontaneous emission via the specific transition enhanced by the cavity when the SiV is not coupled with the cavity. Consequently, the Purcell factor is given by

$$F = \frac{\tau_{\text{off}}/\tau_{\text{on}} - 1}{\xi} + 1.$$

This specific formulation of the Purcell factor has been widely employed across numerous experimental works to accurately account for non-unity quantum efficiency and non-enhanced decay channels [5–7].

To determine a conservative lower bound for the Purcell factor, we must establish the upper bound of ξ. We assume an upper bound of 30% for the quantum efficiency, which is reported to range between 10% and 30% [8–10]. In contrast, the reported Debye-Waller factor exhibits greater consistency, allowing us to use a fixed value of 80% [10, 11]. We also assume that all zero-phonon-line decay is channeled through the transition enhanced by the cavity. This yields an upper bound for ξ of 0.24. Using this value, we calculate the lower bound of the Purcell factor F to be 3.7 ± 0.1.

S7. OPTICAL PROPERTIES OF SIV CENTERS IN THE HETEROGENEOUS PHOTONIC DEVICES

A critical question in heterogeneous photonic integration is whether the process degrades the optical coherence of the embedded color centers. To investigate this, we performed photoluminescence excitation (PLE) spectroscopy (with a laser power of $\sim$0.07 μW power, measured before the objective) on 9 randomly selected SiV centers found within the heterogeneously integrated insertion coupler shown in Fig. 4a of the main text. To capture the long-term stability of these emitters, we monitored the PLE spectra of each SiV center over a 10-minute duration to characterize the spectral diffusion. These results are summarized in Fig. S11.

For the SiV centers in the heterogeneously integrated device, we obtained a mean linewidth of 260 MHz with a standard deviation of 90 MHz. This performance is comparable to previously reported value of 320 ± 180 MHz for SiV centers in bulk diamond [12], and outperforms the linewidth of 410 ± 160 MHz reported for SiV centers in diamond photonic crystals [12]. The spectral diffusion over 10 minutes exhibited a mean range of 120 MHz with a standard deviation of 50 MHz.

As a baseline comparison, Figure S12 shows the PLE spectra for 9 SiV centers measured in the bulk diamond substrate from which the nanobeams were originally fabricated. These bulk emitters were chosen at random and exhibited a mean linewidth of 290 MHz and a standard deviation of 200 MHz. The mean 10-minute spectral diffusion range was 190 MHz with a standard deviation of 120 MHz. The similarity in both the linewidth and the range of spectral diffusion between the heterogeneously integrated and bulk SiV centers indicates that the heterogeneous integration process does not introduce noticeable degradation to the optical coherence properties.

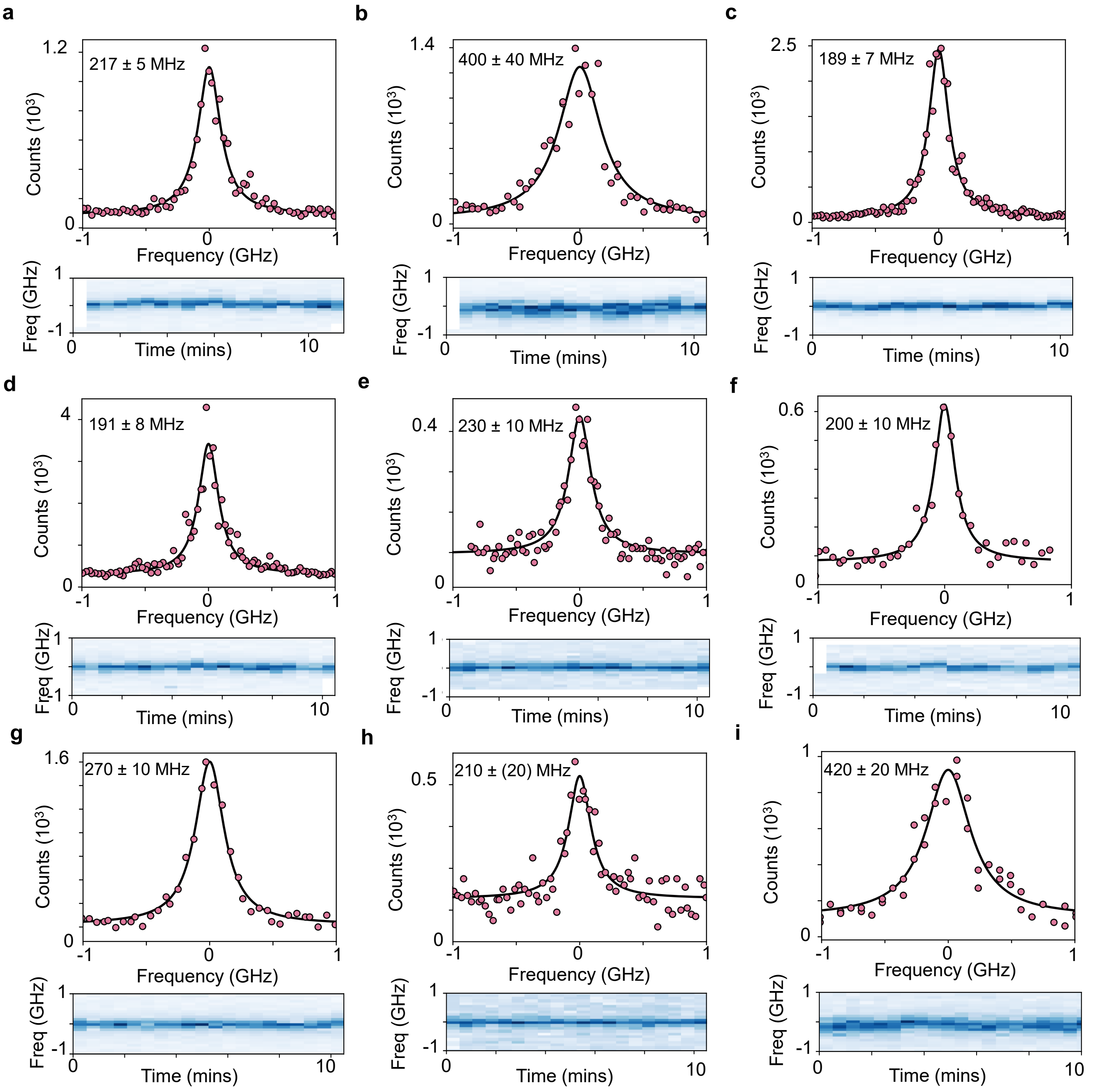

Fig. S11. PLE spectra for 9 randomly selected SiV centers within the heterogeneous photonic device shown in Fig. 4a of the main text. For each emitter, both a representative single-scan spectrum and a 10-minute time-resolved monitoring are shown, from which we extract the emitter linewidth and the range of spectral diffusion in 10 minutes.

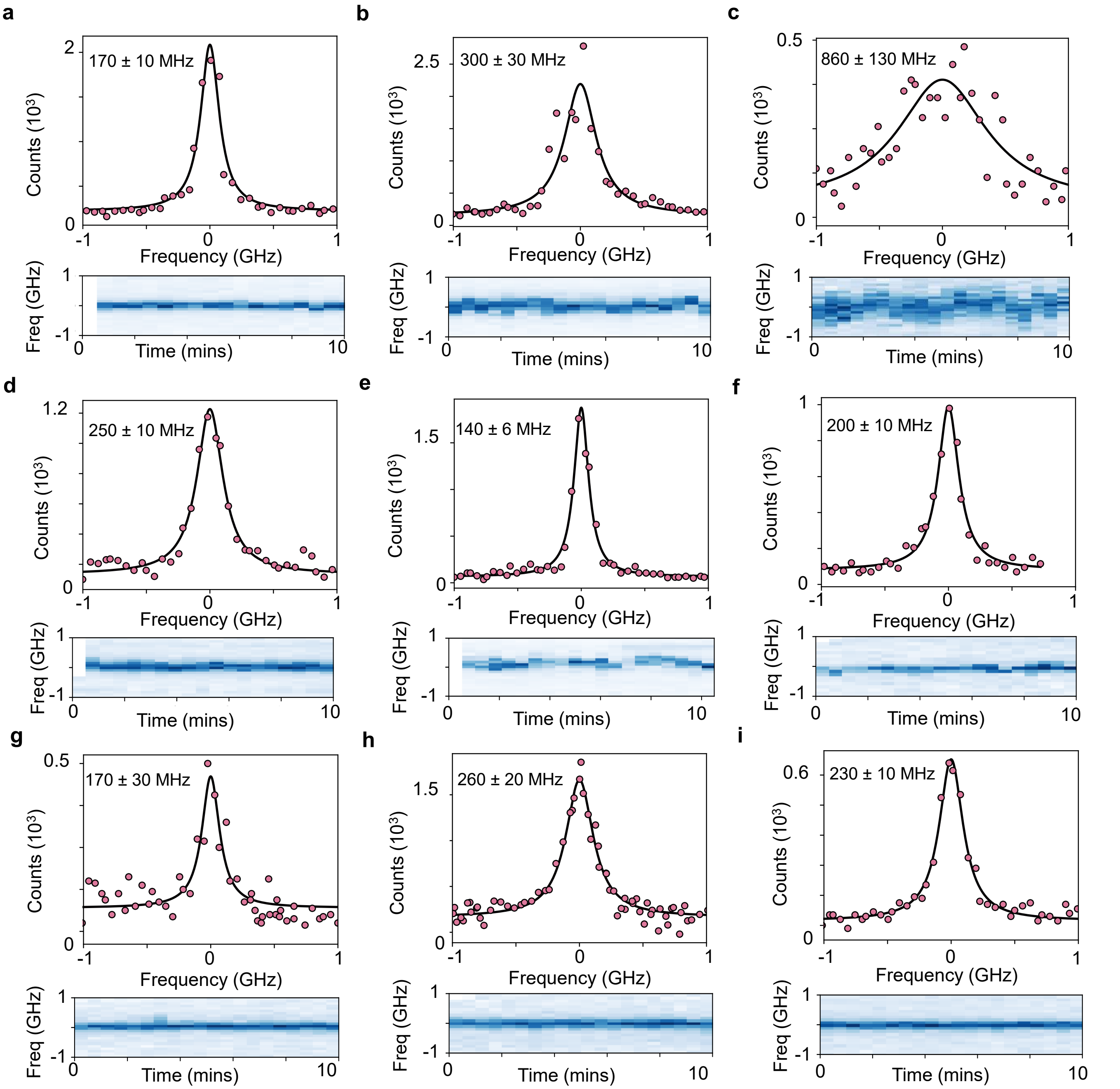

Fig. S12. PLE spectra for 9 randomly selected SiV centers in single crystal bulk diamond. For each emitter, both a representative single-scan spectrum and a 10-minute time-resolved monitoring are shown, from which we extract the emitter linewidth and the range of spectral diffusion in 10 minutes.

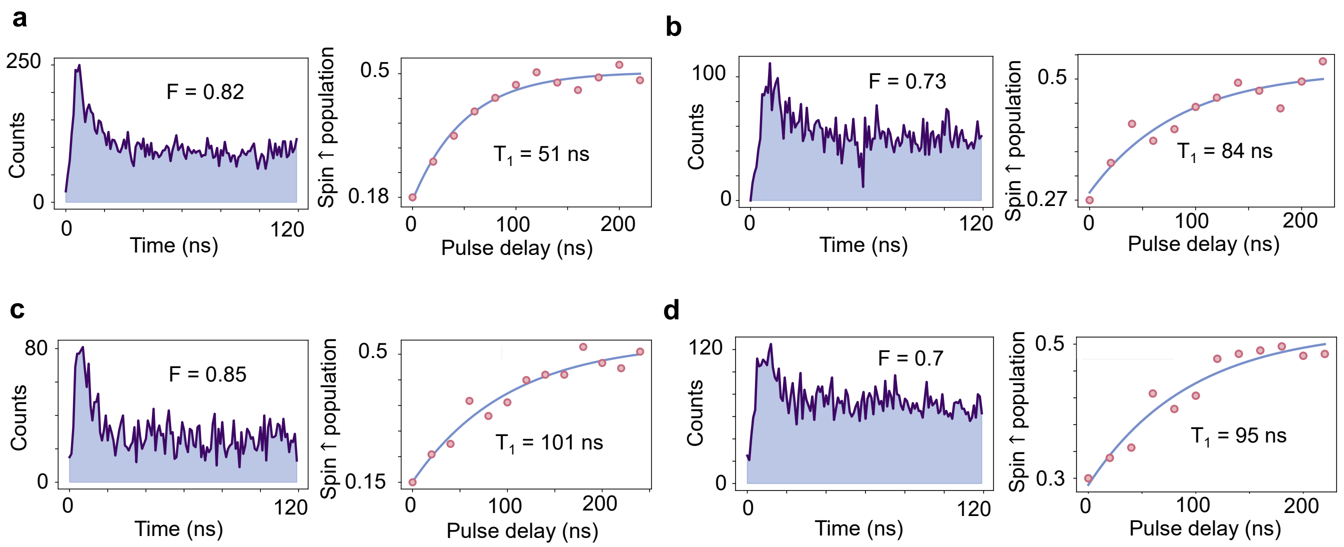

Fig. S13. Measurement of spin initialization fidelity and spin T_1 time for 4 randomly selected SiV centers in single crystal bulk diamond. All pulse sequences and experimental conditions were identical for those used in Fig. 4d and 4e of the main text.

S8. SPIN INITIALIZATION FIDELITY AND T_1 TIME FOR SIV CENTERS IN BULK DIAMOND

Figure S13 shows the spin T_1 time measurements for 4 randomly selected SiV centers in bulk diamond. For each SiV center, we measured the time-resolved photoluminescence under optical pumping to extract the spin initialization fidelity, as well as the spin population revival as a function of the pump-probe delay to determine the spin T_1 time. All pulse sequences and experimental conditions were identical to those used for the results reported in Fig. 4d and 4e of the main text.

We observed spin initialization fidelities ranging from 0.70 to 0.85 and T_1 times between 50 ns and 100 ns. These values are comparable to those reported for the SiV center in the heterogeneously integrated device in Fig. 4 (initialization fidelity of 0.72 and spin T_1 time of 130 ns). Furthermore, these results are consistent with previous report for SiV centers under the same temperature condition (5 K) and magnetic field orientation (along the [001] crystal axis of diamond) [13]. These findings support the conclusion that the heterogeneous integration process does not negatively impact the spin initialization fidelity or the spin T_1 time.

REFERENCES

* dykang@kist.re.kr
* shuosun@colorado.edu

[1] Zhou, X. *et al.* High-efficiency shallow-etched grating on gaas membranes for quantum photonic applications. *Applied physics letters* **113** (2018).
[2] Dory, C. *et al.* Inverse-designed diamond photonics. *Nature communications* **10**, 3309 (2019).
[3] Flexcompute, Inc. Tidy3d, next-gen electromagnetic simulation tool. https://www.flexcompute.com/tidy3d/solver/ (2024).
[4] Butcher, A. *et al.* High-q nanophotonic resonators on diamond membranes using templated atomic layer deposition of tio2. *Nano Letters* **20**, 4603–4609 (2020).
[5] Zhang, J. L. *et al.* Strongly cavity-enhanced spontaneous emission from silicon-vacancy centers in diamond. *Nano letters* **18**, 1360–1365 (2018).
[6] Fehler, K. G. *et al.* Hybrid quantum photonics based on artificial atoms placed inside one hole of a photonic crystal cavity. *ACS photonics* **8**, 2635–2641 (2021).
[7] Ding, S. W. *et al.* Purcell-enhanced emissions from diamond color centers in slow light photonic crystal waveguides. *Nano Letters* **25**, 12125–12131 (2025).

[8] Neu, E., Agio, M. & Becher, C. Photophysics of single silicon vacancy centers in diamond: implications for single photon emission. *Optics express* **20**, 19956–19971 (2012).

[9] Becker, J. N. & Becher, C. Coherence properties and quantum control of silicon vacancy color centers in diamond. *physica status solidi (a)* **214**, 1700586 (2017).

[10] Thiering, G. & Gali, A. Ab initio magneto-optical spectrum of group-iv vacancy color centers in diamond. *Physical Review X* **8**, 021063 (2018).

[11] Neu, E. *et al.* Single photon emission from silicon-vacancy colour centres in chemical vapour deposition nano-diamonds on iridium. *New Journal of Physics* **13**, 025012 (2011).

[12] Evans, R. E., Sipahigil, A., Sukachev, D. D., Zibrov, A. S. & Lukin, M. D. Narrow-linewidth homogeneous optical emitters in diamond nanostructures via silicon ion implantation. *Physical Review Applied* **5**, 044010 (2016).

[13] Pingault, B. *et al.* Coherent control of the silicon-vacancy spin in diamond. *Nature communications* **8**, 15579 (2017).
